# Prognostic Value of the Neutrophil‐to‐Lymphocyte Ratio for All‐Cause Mortality in Patients With Cardiovascular–Kidney–Metabolic Stage 4

**DOI:** 10.1155/mi/9984409

**Published:** 2026-07-27

**Authors:** Longzhou Chen, Wencai Jiang, Guangrong Zhang, Yucai Tang, Hao Guo, Ping Huang, Xuejun Deng

**Affiliations:** ^1^ Department of Cardiology, Suining Central Hospital, Suining, Sichuan, China, sns120.cn

**Keywords:** all-cause mortality, cardiovascular–kidney–metabolic syndrome, CKM Stage 4, critical illness, MIMIC-IV, neutrophil-to-lymphocyte ratio, risk stratification

## Abstract

**Background:**

Cardiovascular–kidney–metabolic (CKM) Stage 4 is defined by established cardiovascular disease in the setting of kidney and/or metabolic dysfunction. Despite the high risk carried by this condition, simple tools for refining prognosis are limited. The neutrophil‐to‐lymphocyte ratio (NLR), derived from routine differential blood counts, has been linked to outcomes in cardiovascular, renal, and critically ill populations. Whether it is prognostically informative in critically ill adults with CKM Stage 4 is not established. We investigated the relationship between NLR and all‐cause mortality in this population.

**Methods:**

Using the Medical Information Mart for Intensive Care IV (MIMIC‐IV), we assembled a retrospective cohort of adults who met CKM Stage 4 criteria. For patients with multiple intensive care unit (ICU) admissions, analysis was restricted to the earliest stay. Admission NLR was derived from the first valid paired neutrophil and lymphocyte measurements recorded within 24 h. Patients were classified by NLR quartile. The prespecified primary endpoints were 30‐day and 365‐day all‐cause mortality, with 90‐day and 180‐day mortality as secondary endpoints. Associations were examined using multivariable Cox regression, restricted cubic splines, subgroup analyses, sensitivity analyses, and mediation analysis.

**Results:**

Among 13,602 eligible patients, mortality rose progressively across NLR quartiles at all assessed time points. After full adjustment, the hazard ratio (HR) for Q4 compared with Q1 was 2.142 (95% confidence interval [CI], 1.858–2.469) for 30‐day mortality. The corresponding 365‐day HR was 1.708 (95% CI, 1.543–1.891). Associations at 90 and 180 days were similar. Restricted cubic splines indicated nonlinear relationships. Results were generally stable in subgroup and sensitivity analyses. Adding NLR improved established ICU severity scores, and blood urea nitrogen (BUN) partially mediated the observed association.

**Conclusions:**

Admission NLR was independently related to higher all‐cause mortality during both short‐ and long‐term follow‐up in critically ill adults with CKM Stage 4. Because it is routinely available, NLR may provide complementary information for risk stratification in this population.

## 1. Introduction

Obesity, hypertension (HTN), chronic kidney disease (CKD), and diabetes mellitus (DM) are major contributors to morbidity and mortality worldwide [1, 2]. These disorders commonly coexist and can reinforce one another through linked cardiovascular, renal, and metabolic pathways. Their coexistence can accelerate disease progression in more than one organ system. In 2023, the American Heart Association (AHA) formalized these interdependencies within the concept of cardiovascular–kidney–metabolic (CKM) syndrome, which subsequently received governmental endorsement [3]. The staging framework spans CKM health Stages 0–4. Stage 4 is the highest‐risk category and requires established cardiovascular disease together with advanced kidney and/or metabolic dysfunction [4].

Patients with CKM Stage 4 nevertheless differ substantially in clinical presentation, disease burden, and prognosis. Patients assigned to the same stage may have different combinations and severities of organ dysfunction. This heterogeneity is clinically important when estimating the individual risk. Their overlapping cardiovascular, kidney, and metabolic abnormalities may also limit the usefulness of tools developed for an individual organ system. Risk stratification therefore requires markers that are readily obtainable and informative across these interacting pathophysiological domains [5, 6].

Inflammation is central to the development and progression of CKM Stage 4 [7]. The neutrophil‐to‐lymphocyte ratio (NLR) is obtained from a routine complete blood count and reflects the balance between neutrophil‐predominant innate activation and lymphocyte‐related adaptive immunity [8–11]. A higher NLR has been associated with all‐cause and cardiovascular mortality in adults with diabetes [12], HTN [13], and the general US population [14]. It has also been associated with CKD progression [15] and with the occurrence and prognosis of acute kidney injury [16]. Its low cost, reproducibility, and routine availability make it clinically practical. NLR can increase through neutrophilia, lymphopenia, or both changes together, linking the measure to systemic stress and immune imbalance. Most evidence, however, comes from single‐disease cohorts or general hospitalized populations. Its prognostic value in the heterogeneous and high‐risk population with CKM Stage 4, therefore, remains uncertain.

Chronic inflammation in CKM Stage 4 commonly accompanies metabolic dysregulation and dysfunction across several organs [17–19]. The mortality gradient across an inflammatory marker may therefore include thresholds or other nonlinear features rather than follow a constant slope. Whether NLR shows a consistent dose‐response association with mortality in CKM Stage 4 is unresolved. Such departures from linearity could contribute to the marked prognostic heterogeneity within this population. The pathophysiological processes that may contribute to this association also require clarification [20–22].

We used Medical Information Mart for Intensive Care IV (MIMIC‐IV) data to examine NLR in relation to all‐cause mortality among critically ill patients with CKM Stage 4. Unlike earlier work in general critical illness or single‐disease cohorts, this study addressed a recently defined condition that integrates cardiovascular, kidney, metabolic, and inflammatory abnormalities. We also tested whether NLR contributed prognostic information beyond the established intensive care unit (ICU) severity scores. Mediation analysis explored a potential pathway involving renal stress. Dose‐response, subgroup, and sensitivity analyses were included to evaluate the robustness and clinical relevance of the findings. The main objective was to determine whether NLR independently identified mortality risk during both short‐ and long‐term follow‐up. Together, these analyses were intended to clarify the role of this simple inflammatory marker in risk stratification.

## 2. Methods

### 2.1. Data Source

All clinical data analyzed within this retrospective secondary analysis originated from version 3.1 of the MIMIC‐IV database, a repository housing anonymized electronic admission records collected at Beth Israel Deaconess Medical Center covering the calendar period spanning 2008–2022 [23]. A locally stored PostgreSQL database instance was queried for eligible clinical information with the assistance of Navicat Premium 16.1.15. The development and public application of the MIMIC‐IV resource received formal ethical clearance from joint institutional review boards affiliated with Beth Israel Deaconess Medical Center and the Massachusetts Institute of Technology. As all individual patient identifiers had been permanently removed from the shared dataset, separate informed consent from enrolled subjects was waived for the present secondary observational research. Human subject research protection training certified by the National Institutes of Health was fully completed by Longzhou Chen, with training certificate ID 74101068 on file.

### 2.2. Study Design and Population

**Figure 1:**
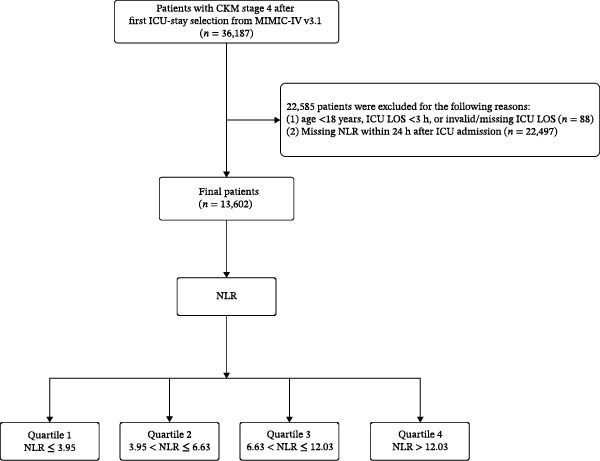
Flow of cohort assembly. ICU, intensive care unit; MIMIC‐IV, Medical Information Mart for Intensive Care IV; NLR, neutrophil‐to‐lymphocyte ratio.

CKM Stage 4 was defined according to the AHA Presidential Advisory [4]. We analyzed each patient’s earliest qualifying ICU admission. Inclusion required age ≥18 years and an ICU stay of at least 3 h. Patients also needed established clinical cardiovascular disease, at least one CKM risk factor, and paired neutrophil and lymphocyte counts obtained within 24 h of ICU admission. Cardiovascular disease was identified from ICD‐9 and ICD‐10 codes for coronary heart disease (CHD) or myocardial infarction (MI), heart failure (HF), atrial fibrillation (AF), stroke or transient ischemic attack, and peripheral vascular disease (PVD). The coded CKM risk factors were DM, HTN, CKD, obesity, and dyslipidemia. Initial screening identified 36,187 patients with CKM Stage 4. We removed 88 patients who were younger than 18 years, remained in the ICU for less than 3 h, or had missing or invalid length‐of‐stay data. Among the remaining 36,099 patients, 22,497 had no valid paired neutrophil and lymphocyte measurements during the first 24 h. The final analytic cohort comprised 13,602 patients (Figure 1).

### 2.3. Variable Extraction and Processing

Structured query language (SQL) scripts executed in Navicat Premium 16.1.15 were used to retrieve data from MIMIC‐IV v3.1. Demographic variables comprised age, sex, race, insurance status, marital status, height, and weight. The first 24 h after ICU admission provided heart rate, systolic blood pressure (SBP), diastolic blood pressure (DBP), mean arterial pressure (MAP), temperature, respiratory rate (RR), and peripheral oxygen saturation (SpO_2_). Blood count variables from the same period included hemoglobin (Hb), hematocrit, red and white blood cell counts, platelet count (PLT), red cell distribution width, absolute lymphocyte count (ALC), absolute monocyte count (AMC), and absolute neutrophil count (ANC). Other laboratory variables included albumin, lactate, blood glucose (GLU), glycated Hb (HbA1c), triglycerides, blood urea nitrogen (BUN), serum creatinine (Scr), sodium (Na), potassium (K), chloride (Cl), calcium (Ca), bicarbonate, anion gap, international normalized ratio, prothrombin time, activated partial thromboplastin time, and C‐reactive protein. Illness severity was represented by the Sequential Organ Failure Assessment (SOFA), Simplified Acute Physiology Score II (SAPS II), Acute Physiology Score III (APS III), and Oxford Acute Severity of Illness Score (OASIS). ICD‐9 and ICD‐10 codes identified HTN, DM, AF, acute kidney injury, pneumonia, cerebrovascular disease (CVA), HF, MI, ischemic heart disease, PVD, CKD, obesity, and dyslipidemia. Treatment variables were mechanical ventilation, vasopressor and insulin use, renal replacement therapy, continuous renal replacement therapy, corticosteroids, statins, sodium‐GLU cotransporter‐2 inhibitors, and glucagon‐like peptide‐1 receptor agonists. For vital signs and laboratory tests, the earliest nonmissing value was selected. Neutrophil and lymphocyte counts had to come from the same differential count. Patients without such a pair were excluded from the primary NLR analysis. First‐day SOFA, SAPS II, APS III, and OASIS values followed established MIMIC‐Code algorithms.

### 2.4. NLR Definition and Outcomes

For each patient, NLR equaled the ANC divided by the ALC [24]. Quartile cut points in the analytic cohort defined Q1 (NLR ≤ 3.95), Q2 (3.95 < NLR ≤ 6.63), Q3 (6.63 < NLR ≤ 12.03), and Q4 (NLR > 12.03). Death from any cause within 30 and 365 days of ICU admission was evaluated as the primary outcome. The corresponding 90‐ and 180‐day endpoints were secondary outcomes. Sensitivity analyses used NLR at 48–72 h and the change from the first‐24‐h measurement to the 48–72‐h measurement.

### 2.5. Management of Missing Data and Outliers

Covariates with more than 20% of missing observations were excluded from multivariable modeling (Table S1). For variables below this threshold, missing values were imputed in Python with the scikit‐learn random forest procedure. Five datasets were completed using distinct random seeds, and regression estimates were pooled according to Rubin’s rules. NLR, follow‐up duration, and mortality status were retained as observed.

### 2.6. Statistical Methods

We summarized baseline variables by the NLR quartile and separately by survival status at 30, 90, 180, and 365 days. Continuous data are expressed as mean ± standard deviation or median (interquartile range) depending on distribution. Group comparisons used one‐way analysis of variance or the Kruskal–Wallis test. Categorical data are given as counts and percentages and were compared by Pearson’s chi‐square test or, when appropriate, Fisher’s exact test. To examine selection related to missing leukocyte data, we also compared included patients with eligible patients who lacked a valid neutrophil or lymphocyte count during the first 24 h after ICU admission.

We estimated hazard ratios (HRs) for all‐cause death using Cox proportional hazards regression. Quartile analyses used Q1 as the reference, whereas continuous analyses transformed NLR into ln(NLR + 1) because of right skew. Three levels of adjustment were prespecified. Model 1 contained no covariates. Model 2 included age, sex, and race. Model 3 additionally incorporated vital signs, laboratory measurements, comorbidities, corticosteroid use, and statin use.

Restricted cubic spline terms were added to Model 3 to evaluate the shape of the NLR‐mortality association. Kaplan–Meier methods estimated survival in each NLR quartile, and the log‐rank test compared the curves. Prespecified subgroup analyses considered age, sex, race, HTN, DM, HF, CKD, obesity, and dyslipidemia. Interaction terms tested whether the association differed across these strata.

Discrimination and calibration were examined with receiver operating characteristic (ROC) curves, AUC, C‐index, and calibration plots. The incremental contribution of the NLR was evaluated by adding it separately to SOFA, SAPS II, APS III, and OASIS. Additional analyses considered NLR measured at 48‐72 h, the change in NLR over time, and mediation by BUN. Scr and eGFR were used as alternative mediators in the sensitivity analyses.

Statistical analyses were run in Python 3.11.9 with pandas 2.3.3, NumPy 2.3.4, SciPy 1.16.2, scikit‐learn 1.7.2, lifelines 0.30.0, and matplotlib 3.10.7. All tests were two‐sided, and *p*  < 0.05 was considered statistically significant.

## 3. Results

### 3.1. Characteristics of the Study Cohort

The analytic cohort contained 13,602 patients (Table 1). The median age was 70.00 years (IQR, 61.00–78.00), and 8640 patients (63.5%) were male. Across progressively higher NLR quartiles, age, heart rate, RR, WBC, ANC, BUN, Scr, GLU, and severity scores increased, whereas ALC and SpO_2_ decreased. The prevalence of CKD, AF, HF, CVA, and PVD also differed among quartiles. Tables 2 and 3 compare patients by the survival status. There were 2063 deaths by day 30 and 3620 by day 365. NLR was higher among nonsurvivors at both 30 days (11.75 [6.52–22.73] vs. 6.11 [3.78–10.52], *p*  < 0.001) and 365 days (10.36 [5.41–19.84] vs. 5.86 [3.70–9.85], *p*  < 0.001). Q4 contained 48.86% of 30‐day deaths and 43.29% of 365‐day deaths. Nonsurvivors also had poorer renal indices and higher SOFA, SAPS II, APS III, and OASIS scores. Results for 90‐day and 180‐day survival status appear in Tables S2 and S3.

**Table 1:** Patient characteristics across NLR quartiles.

Characteristic	Overall (*n* = 13,602)	Q1 (*n* = 3401)	Q2 (*n* = 3400)	Q3 (*n* = 3400)	Q4 (*n* = 3401)	*p* value
Demographics						
Age (years)	70.00 (61.00, 78.00)	69.00 (60.00, 77.00)	68.00 (60.00, 76.00)	70.00 (61.00, 79.00)	72.00 (63.00, 81.00)	<0.001
Male sex	8640 (63.5)	2104 (61.9)	2343 (68.9)	2191 (64.4)	2002 (58.9)	<0.001
Race: White	9057 (66.6)	2196 (64.6)	2259 (66.4)	2337 (68.7)	2265 (66.6)	<0.001
Race: Black	987 (7.3)	309 (9.1)	230 (6.8)	215 (6.3)	233 (6.9)	—
Race: other	3558 (26.2)	896 (26.3)	911 (26.8)	848 (24.9)	903 (26.6)	—
Vital signs						
HR (beats/min)	82.00 (74.00, 95.00)	80.00 (72.00, 88.00)	80.00 (73.00, 88.00)	84.00 (75.00, 96.00)	90.00 (77.00, 105.00)	<0.001
SBP (mm Hg)	117.00 (104.00, 134.00)	116.00 (103.00, 131.00)	117.00 (104.00, 131.00)	118.00 (104.00, 135.00)	120.00 (104.00, 138.00)	<0.001
DBP (mm Hg)	63.00 (54.00, 74.00)	62.00 (53.00, 71.00)	62.00 (53.00, 71.00)	63.00 (54.00, 75.00)	65.00 (55.00, 78.00)	<0.001
RR (breaths/min)	18.00 (15.00, 22.00)	16.00 (15.00, 19.00)	16.00 (15.00, 20.00)	18.00 (15.00, 22.00)	20.00 (16.00, 24.00)	<0.001
SpO_2_ (%)	99.00 (96.00, 100.00)	100.00 (97.00, 100.00)	100.00 (97.00, 100.00)	99.00 (96.00, 100.00)	97.00 (94.00, 100.00)	<0.001
Laboratory parameters						
NLR	6.63 (3.95, 12.03)	2.80 (2.06, 3.40)	5.13 (4.52, 5.84)	8.69 (7.56, 10.07)	20.38 (15.16, 30.56)	<0.001
ANC (K/µL)	9.27 (6.35, 13.04)	5.97 (3.88, 8.15)	8.69 (6.46, 11.31)	10.35 (7.64, 13.44)	13.35 (9.72, 18.33)	<0.001
ALC (K/µL)	1.32 (0.80, 2.01)	2.20 (1.54, 3.03)	1.69 (1.25, 2.23)	1.18 (0.86, 1.56)	0.62 (0.40, 0.88)	<0.001
WBC (K/µL)	11.70 (8.40, 15.90)	9.00 (6.30, 12.40)	11.10 (8.40, 14.40)	12.40 (9.20, 16.10)	15.00 (11.00, 20.50)	<0.001
Hb (g/dL)	10.10 (8.60, 11.60)	9.60 (8.20, 11.10)	9.90 (8.50, 11.40)	10.30 (8.90, 11.90)	10.40 (9.00, 12.00)	<0.001
PLT (K/µL)	165.00 (122.00, 227.00)	144.00 (111.00, 193.00)	155.00 (121.00, 207.00)	175.00 (129.00, 239.00)	197.00 (139.00, 269.25)	<0.001
BUN (mg/dL)	20.00 (14.00, 32.00)	17.00 (13.00, 24.00)	17.00 (13.00, 25.00)	21.00 (15.00, 34.00)	28.00 (18.00, 47.00)	<0.001
Scr (mg/dL)	1.00 (0.80, 1.50)	0.90 (0.70, 1.20)	0.90 (0.70, 1.30)	1.10 (0.80, 1.60)	1.30 (0.90, 2.10)	<0.001
Glu (mg/dL)	126.00 (107.00, 158.00)	119.00 (103.00, 141.00)	121.00 (105.00, 145.00)	129.00 (108.00, 163.00)	143.00 (113.00, 192.00)	<0.001
Na (mmol/L)	138.00 (136.00, 141.00)	138.00 (136.00, 141.00)	138.00 (136.00, 141.00)	138.00 (136.00, 141.00)	138.00 (135.00, 141.00)	<0.001
K (mmol/L)	4.30 (3.90, 4.70)	4.20 (3.90, 4.60)	4.30 (3.90, 4.60)	4.30 (3.90, 4.70)	4.20 (3.80, 4.80)	0.004
Comorbidities						
HTN	11,587 (85.2)	2926 (86.0)	2895 (85.1)	2908 (85.5)	2858 (84.0)	0.120
DM	5425 (39.9)	1422 (41.8)	1351 (39.7)	1322 (38.9)	1330 (39.1)	0.056
CKD	3677 (27.0)	784 (23.1)	792 (23.3)	940 (27.6)	1161 (34.1)	<0.001
AF	6414 (47.2)	1430 (42.0)	1603 (47.1)	1631 (48.0)	1750 (51.5)	<0.001
HF	5349 (39.3)	1055 (31.0)	1150 (33.8)	1457 (42.9)	1687 (49.6)	<0.001
MI	3587 (26.4)	891 (26.2)	909 (26.7)	916 (26.9)	871 (25.6)	0.600
IHD	8784 (64.6)	2371 (69.7)	2380 (70.0)	2146 (63.1)	1887 (55.5)	<0.001
CVA	2324 (17.1)	647 (19.0)	508 (14.9)	592 (17.4)	577 (17.0)	<0.001
PVD	2320 (17.1)	519 (15.3)	567 (16.7)	607 (17.9)	627 (18.4)	0.003
Disease severity and ICU treatments						
SOFA	5.00 (3.00, 7.00)	5.00 (3.00, 7.00)	5.00 (3.00, 7.00)	5.00 (3.00, 7.00)	6.00 (3.00, 8.00)	<0.001
SAPS II	38.00 (31.00, 47.00)	36.00 (29.00, 44.00)	36.00 (30.00, 43.00)	38.00 (31.00, 47.00)	43.00 (35.00, 53.00)	<0.001
APS III	41.00 (31.00, 57.00)	36.00 (27.00, 49.00)	36.00 (28.00, 49.00)	42.00 (32.00, 56.00)	52.00 (39.00, 68.00)	<0.001
OASIS	32.00 (27.00, 38.00)	31.00 (26.00, 36.00)	31.00 (26.00, 37.00)	33.00 (28.00, 39.00)	35.00 (29.00, 42.00)	<0.001
Mechanical ventilation	8938 (65.7)	2348 (69.0)	2519 (74.1)	2191 (64.4)	1880 (55.3)	<0.001
RRT	726 (5.3)	107 (3.1)	116 (3.4)	199 (5.9)	304 (8.9)	<0.001
Vasopressor use	6930 (50.9)	1790 (52.6)	1850 (54.4)	1683 (49.5)	1607 (47.3)	<0.001
Medication use during hospitalization						
Corticosteroids	2747 (20.2)	447 (13.1)	426 (12.5)	690 (20.3)	1184 (34.8)	<0.001
Statins	9518 (70.0)	2616 (76.9)	2634 (77.5)	2357 (69.3)	1911 (56.2)	<0.001
SGLT2 inhibitors	40 (0.3)	5 (0.1)	12 (0.4)	14 (0.4)	9 (0.3)	0.202
GLP‐1 receptor agonists	2 (0.0)	1 (0.0)	0 (0.0)	1 (0.0)	0 (0.0)	0.572

*Note:* K, serum potassium; Na, serum sodium; PLT, platelet count; Scr, serum creatinine; SpO_2_, peripheral oxygen saturation; WBC, white blood cell count.

Abbreviations: AF, atrial fibrillation; ALC, absolute lymphocyte count; ANC, absolute neutrophil count; APS III, Acute Physiology Score III; BUN, blood urea nitrogen; CKD, chronic kidney disease; CVA, cerebrovascular disease; DBP, diastolic blood pressure; DM, diabetes mellitus; GLU, blood glucose; Hb, hemoglobin; HF, heart failure; HR, heart rate; HTN, hypertension; IHD, ischemic heart disease; MI, myocardial infarction; NLR, neutrophil‐to‐lymphocyte ratio; OASIS, Oxford Acute Severity of Illness Score; PVD, peripheral vascular disease; RR, respiratory rate; RRT, renal replacement therapy; SAPS II, Simplified Acute Physiology Score II; SBP, systolic blood pressure; SOFA, Sequential Organ Failure Assessment.

**Table 2:** Clinical characteristics by 30‐day survival status.

Characteristics	Overall (*n* = 13,602)	Survivors (*n* = 11,539)	Nonsurvivors (*n* = 2063)	*p* value
Age (years)	70.00 (61.00–78.00)	69.00 (60.00–77.00)	75.00 (66.00–83.00)	<0.001
Sex (%)				<0.001
Female	4962 (36.48)	4039 (35.00)	923 (44.74)	—
Male	8640 (63.52)	7500 (65.00)	1140 (55.26)	—
Race (%)				<0.001
White	9057 (66.59)	7815 (67.73)	1242 (60.20)	—
Black	987 (7.26)	833 (7.22)	154 (7.46)	—
Other	3558 (26.16)	2891 (25.05)	667 (32.33)	—
NLR	6.63 (3.95–12.03)	6.11 (3.78–10.52)	11.75 (6.52–22.73)	<0.001
NLR quartile (%)				<0.001
Q1	3401 (25.00)	3125 (27.08)	276 (13.38)	—
Q2	3400 (25.00)	3150 (27.30)	250 (12.12)	—
Q3	3400 (25.00)	2871 (24.88)	529 (25.64)	—
Q4	3401 (25.00)	2393 (20.74)	1008 (48.86)	—
HR (beats/min)	82.00 (74.00–95.00)	80.00 (74.00–92.00)	91.00 (77.00–107.00)	<0.001
SBP (mm Hg)	117.00 (104.00–134.00)	118.00 (104.00–134.00)	117.00 (101.00–136.00)	0.055
DBP (mm Hg)	63.00 (54.00–74.00)	62.00 (54.00–73.00)	64.00 (53.00–78.00)	0.001
RR (breaths/min)	18.00 (15.00–22.00)	17.00 (15.00–21.00)	21.00 (17.00–25.00)	<0.001
SpO_2_ (%)	99.00 (96.00–100.00)	99.00 (96.00–100.00)	97.00 (94.00–100.00)	<0.001
ANC (K/µL)	9.27 (6.35–13.04)	9.04 (6.29–12.53)	10.76 (6.87–16.29)	<0.001
ALC (K/µL)	1.32 (0.80–2.01)	1.41 (0.88–2.10)	0.86 (0.50–1.39)	<0.001
WBC (K/µL)	11.70 (8.40–15.90)	11.50 (8.40–15.40)	12.90 (8.70–19.20)	<0.001
Hb (g/dL)	10.10 (8.60–11.60)	10.00 (8.60–11.60)	10.20 (8.60–11.90)	0.006
PLT (K/µL)	165.00 (122.00–227.00)	163.00 (122.00–221.00)	186.00 (120.00–265.00)	<0.001
BUN (mg/dL)	20.00 (14.00–32.00)	19.00 (14.00–29.00)	32.00 (20.00–53.00)	<0.001
Scr (mg/dL)	1.00 (0.80–1.50)	1.00 (0.80–1.40)	1.50 (1.00–2.30)	<0.001
GLU (mg/dL)	126.00 (107.00–158.00)	125.00 (106.00–153.00)	142.00 (109.00–195.00)	<0.001
Na (mmol/L)	138.00 (136.00–141.00)	138.00 (136.00–141.00)	139.00 (135.00–142.00)	0.057
K (mmol/L)	4.30 (3.90–4.70)	4.20 (3.90–4.60)	4.30 (3.80–4.80)	0.031
SOFA	5.00 (3.00–7.00)	5.00 (3.00–7.00)	7.00 (4.00–10.00)	<0.001
SAPS II	38.00 (31.00–47.00)	36.00 (30.00–44.00)	51.00 (40.00–62.00)	<0.001
APS III	41.00 (31.00–57.00)	38.00 (29.00–51.00)	62.00 (47.00–82.00)	<0.001
OASIS	32.00 (27.00–38.00)	32.00 (26.00–37.00)	39.00 (33.00–45.00)	<0.001
HTN (%)	11,587 (85.19)	9844 (85.31)	1743 (84.49)	0.350
DM (%)	5425 (39.88)	4616 (40.00)	809 (39.21)	0.516
CKD (%)	3677 (27.03)	2937 (25.45)	740 (35.87)	<0.001
Obesity (%)	2935 (21.58)	2488 (21.56)	447 (21.67)	0.937
Dyslipidemia (%)	8724 (64.14)	7670 (66.47)	1054 (51.09)	<0.001
AF (%)	6414 (47.15)	5303 (45.96)	1111 (53.85)	<0.001
HF (%)	5349 (39.33)	4347 (37.67)	1002 (48.57)	<0.001
MI (%)	3587 (26.37)	3014 (26.12)	573 (27.78)	0.123
IHD (%)	8784 (64.58)	7684 (66.59)	1100 (53.32)	<0.001
CVA (%)	2324 (17.09)	1805 (15.64)	519 (25.16)	<0.001
PVD (%)	2320 (17.06)	1923 (16.67)	397 (19.24)	0.005
Mechanical ventilation (%)	8938 (65.71)	7569 (65.59)	1369 (66.36)	0.516
RRT (%)	726 (5.34)	450 (3.90)	276 (13.38)	<0.001
Vasopressor use (%)	6930 (50.95)	5778 (50.07)	1152 (55.84)	<0.001
Corticosteroid use (%)	2747 (20.20)	1997 (17.31)	750 (36.35)	<0.001
Statin use (%)	9518 (69.98)	8599 (74.52)	919 (44.55)	<0.001

*Note:* K, serum potassium; Na, serum sodium; PLT, platelet count; Scr, serum creatinine; SpO_2_, peripheral oxygen saturation; WBC, white blood cell count.

Abbreviations: AF, atrial fibrillation; ALC, absolute lymphocyte count; ANC, absolute neutrophil count; APS III, Acute Physiology Score III; BUN, blood urea nitrogen; CKD, chronic kidney disease; CVA, cerebrovascular disease; DBP, diastolic blood pressure; DM, diabetes mellitus; GLU, blood glucose; Hb, hemoglobin; HF, heart failure; HR, heart rate; HTN, hypertension; IHD, ischemic heart disease; MI, myocardial infarction; NLR, neutrophil‐to‐lymphocyte ratio; OASIS, Oxford Acute Severity of Illness Score; PVD, peripheral vascular disease; RR, respiratory rate; RRT, renal replacement therapy; SAPS II, Simplified Acute Physiology Score II; SBP, systolic blood pressure; SOFA, Sequential Organ Failure Assessment.

**Table 3:** Clinical characteristics by 365‐day survival status.

Characteristic	Overall (*n* = 13,602)	Survivors (*n* = 9982)	Nonsurvivors (*n* = 3620)	*p* value
Demographic characteristics				
Age (years)	70.00 (61.00–78.00)	68.00 (60.00–76.00)	75.00 (66.00–83.00)	<0.001
Sex (%)				<0.001
Female	4962 (36.48)	3381 (33.87)	1581 (43.67)	—
Male	8640 (63.52)	6601 (66.13)	2039 (56.33)	—
Race (%)				0.004
White	9057 (66.59)	6705 (67.17)	2352 (64.97)	—
Black	987 (7.26)	683 (6.84)	304 (8.40)	—
Other	3558 (26.16)	2594 (25.99)	964 (26.63)	—
NLR	6.63 (3.95–12.03)	5.86 (3.70–9.85)	10.36 (5.41–19.84)	<0.001
NLR quartile (%)				<0.001
Q1	3401 (25.00)	2810 (28.15)	591 (16.33)	—
Q2	3400 (25.00)	2858 (28.63)	542 (14.97)	—
Q3	3400 (25.00)	2480 (24.84)	920 (25.41)	—
Q4	3401 (25.00)	1834 (18.37)	1567 (43.29)	—
HR (beats/min)	82.00 (74.00–95.00)	80.00 (74.00–91.00)	89.00 (76.00–105.00)	<0.001
SBP (mmHg)	117.00 (104.00–134.00)	117.00 (104.00–133.00)	119.00 (102.00–137.00)	0.090
DBP (mmHg)	63.00 (54.00–74.00)	62.00 (54.00–73.00)	64.00 (53.00–78.00)	<0.001
RR (breaths/min)	18.00 (15.00–22.00)	16.50 (15.00–20.00)	20.00 (16.00–24.00)	<0.001
SpO_2_ (%)	99.00 (96.00–100.00)	99.00 (96.00–100.00)	97.00 (94.00–100.00)	<0.001
ANC (K/µL)	9.27 (6.35–13.04)	9.08 (6.37–12.45)	9.78 (6.23–14.95)	<0.001
ALC (K/µL)	1.32 (0.80–2.01)	1.49 (0.95–2.18)	0.91 (0.53–1.44)	<0.001
WBC (K/µL)	11.70 (8.40–15.90)	11.60 (8.50–15.40)	12.10 (8.10–17.60)	<0.001
Hb (g/dL)	10.10 (8.60–11.60)	10.10 (8.60–11.60)	10.10 (8.60–11.70)	0.631
PLT (K/µL)	165.00 (122.00–227.00)	161.00 (122.00–216.00)	185.00 (122.75–262.00)	<0.001
BUN (mg/dL)	20.00 (14.00–32.00)	18.00 (13.00–26.00)	31.00 (19.00–50.00)	<0.001
Scr (mg/dL)	1.00 (0.80–1.50)	0.90 (0.80–1.30)	1.40 (0.90–2.20)	<0.001
GLU (mg/dL)	126.00 (107.00–158.00)	124.00 (106.00–151.00)	136.00 (108.00–185.00)	<0.001
Na (mmol/L)	138.00 (136.00–141.00)	138.00 (136.00–141.00)	138.00 (135.00–142.00)	0.731
K (mmol/L)	4.30 (3.90–4.70)	4.30 (3.90–4.60)	4.30 (3.80–4.80)	0.196
SOFA	5.00 (3.00–7.00)	5.00 (3.00–7.00)	6.00 (4.00–9.00)	<0.001
SAPS II	38.00 (31.00–47.00)	36.00 (29.00–43.00)	47.00 (38.00–57.00)	<0.001
APS III	41.00 (31.00–57.00)	37.00 (28.00–49.00)	56.00 (42.00–74.00)	<0.001
OASIS	32.00 (27.00–38.00)	31.00 (26.00–36.00)	37.00 (31.00–43.00)	<0.001
HTN	11,587 (85.19)	8547 (85.62)	3040 (83.98)	0.018
DM	5425 (39.88)	3926 (39.33)	1499 (41.41)	0.030
CKD	3677 (27.03)	2304 (23.08)	1373 (37.93)	<0.001
Obesity	2935 (21.58)	2140 (21.44)	795 (21.96)	0.528
Dyslipidemia	8724 (64.14)	6855 (68.67)	1869 (51.63)	<0.001
AF	6414 (47.15)	4445 (44.53)	1969 (54.39)	<0.001
HF	5349 (39.33)	3475 (34.81)	1874 (51.77)	<0.001
MI	3587 (26.37)	2617 (26.22)	970 (26.80)	0.513
IHD	8784 (64.58)	6783 (67.95)	2001 (55.28)	<0.001
CVA	2324 (17.09)	1503 (15.06)	821 (22.68)	<0.001
PVD	2320 (17.06)	1625 (16.28)	695 (19.20)	<0.001
Mechanical ventilation	8938 (65.71)	6862 (68.74)	2076 (57.35)	<0.001
RRT	726 (5.34)	306 (3.07)	420 (11.60)	<0.001
Vasopressor use	6930 (50.95)	5183 (51.92)	1747 (48.26)	<0.001

*Note:* K, serum potassium; Na, serum sodium; PLT, platelet count; Scr, serum creatinine; SpO_2_, peripheral oxygen saturation; WBC, white blood cell count.

Abbreviations: AF, atrial fibrillation; ALC, absolute lymphocyte count; ANC, absolute neutrophil count; APS III, Acute Physiology Score III; BUN, blood urea nitrogen; CKD, chronic kidney disease; CVA, cerebrovascular disease; DBP, diastolic blood pressure; DM, diabetes mellitus; GLU, blood glucose; Hb, hemoglobin; HF, heart failure; HR, heart rate; HTN, hypertension; IHD, ischemic heart disease; MI, myocardial infarction; NLR, neutrophil‐to‐lymphocyte ratio; OASIS, Oxford Acute Severity of Illness Score; PVD, peripheral vascular disease; RR, respiratory rate; RRT, renal replacement therapy; SAPS II, Simplified Acute Physiology Score II; SBP, systolic blood pressure; SOFA, Sequential Organ Failure Assessment.

### 3.2. Collinearity Assessment for Model 3

We evaluated collinearity in Model 3 with generalized variance inflation factors (GVIFs). The complete case design matrix contained 13,443 patients. Every adjusted measure [GVIF^(1/(2−Df))^] was below 1.5 (Table S4), indicating that substantial multicollinearity was unlikely. The largest adjusted values were 1.4915 for BUN and 1.4712 for Scr. NLR had a GVIF of 1.1583 and an adjusted value of 1.0763. These results supported the stability of the fully adjusted estimates.

### 3.3. Survival Across NLR Quartiles

**Figure 2:**
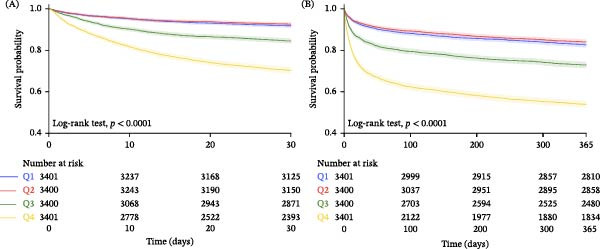
Kaplan–Meier survival estimates by NLR quartile. Subpart (A) shows the 30‐day endpoint and Subpart (B) shows the 365‐day endpoint. Q1 was NLR ≤ 3.95; Q2, 3.95 < NLR ≤ 6.63; Q3, 6.63 < NLR ≤ 12.03; Q4, NLR > 12.03. The curves were compared with the log‐rank test.

Among patients with CKM Stage 4, survival differed among the four NLR groups (log‐rank *p*  < 0.001). At both 30 and 365 days, the estimated survival probability decreased from Q1 to Q4. Patients in Q4 (NLR > 12.03) had the poorest survival, while the lower quartiles had progressively more favorable curves (Figure 2A,B). Separation was evident shortly after ICU admission and persisted throughout follow‐up.

### 3.4. Associations of NLR With Mortality in CKM Stage 4

Among patients with CKM Stage 4, Model 3 showed higher mortality with increasing NLR. Relative to Q1 (NLR ≤ 3.95), Q3 and Q4 had higher 30‐day risks (Q3: HR 1.538, 95% CI 1.324–1.786; Q4: HR 2.142, 95% CI 1.858–2.469). The same pattern was present at 365 days (Q3: HR 1.260, 95% CI 1.133–1.401; Q4: HR 1.708, 95% CI 1.543–1.891). Trends across quartiles were significant for both 30‐ and 365‐day mortality (both *p* for trend <0.001; Table 4). Results at 90 and 180 days were comparable. In Model 3, Q4 versus Q1 had an HR of 1.969 (95% CI 1.745–2.223) for 90‐day mortality and 1.841 (95% CI 1.647–2.058) for 180‐day mortality. Both additional endpoints also showed significant quartile trends (both *p* for trend <0.001; Table S5).

**Table 4:** Cox models for 30‐day and 365‐day mortality across NLR quartiles.

Events (%)		Model 1		Model 2		Model 3	
		HR (95% CI)	*p* value	HR (95% CI)	*p* value	HR (95% CI)	*p* value
NLR quartile							
30‐day mortality							
Q1 (*n* = 3401)	276 (8.1)	1	—	1	—	1	—
Q2 (*n* = 3400)	250 (7.4)	0.902 (0.761–1.071)	0.239	0.922 (0.777–1.094)	0.351	0.925 (0.778–1.099)	0.374
Q3 (*n* = 3400)	529 (15.6)	1.994 (1.725–2.306)	<0.001	1.942 (1.679–2.247)	<0.001	1.538 (1.324–1.786)	<0.001
Q4 (*n* = 3401)	1008 (29.6)	4.125 (3.612–4.712)	<0.001	3.804 (3.328–4.348)	<0.001	2.142 (1.858–2.469)	<0.001
*p* for trend	—	—	<0.001	—	<0.001	—	<0.001
365‐day mortality							
Q1 (*n* = 3401)	591 (17.4)	1	—	1	—	1	—
Q2 (*n* = 3400)	542 (15.9)	0.911 (0.810–1.023)	0.115	0.934 (0.831–1.050)	0.251	0.913 (0.812–1.027)	0.129
Q3 (*n* = 3400)	920 (27.1)	1.670 (1.506–1.852)	<0.001	1.618 (1.459–1.795)	<0.001	1.260 (1.133–1.401)	<0.001
Q4 (*n* = 3401)	1567 (46.1)	3.308 (3.009–3.636)	<0.001	3.032 (2.757–3.335)	<0.001	1.708 (1.543–1.891)	<0.001
*p* for trend	—	—	<0.001	—	<0.001	—	<0.001

Model 1 contained no adjustment variables. Model 2 accounted for age, sex, and race. Model 3 used the full clinical covariate set specified in Section 2.6 and detailed in the Table 4 footnote.

**Figure 3:**
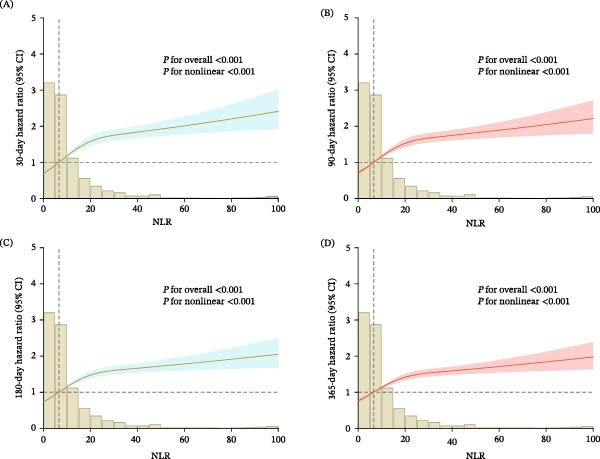
NLR distributions and restricted cubic spline estimates for all‐cause mortality at 30 (A), 90 (B), 180 (C), and 365 days (D). Hazard ratios (HRs) were estimated with Model 3 in the CKM Stage 4 cohort, using the covariates specified in Section 2.6. Curves show HRs, shaded areas indicate 95% confidence intervals, and histograms display the NLR distribution. The cohort median was the reference value.

Spline models were fitted to determine whether mortality changed linearly across the NLR range. Model 3 showed nonlinear dose‐response patterns for all‐cause mortality at 30, 90, 180, and 365 days. Each test for nonlinearity yielded *p*  < 0.001 (Figure 3).

### 3.5. Model Discrimination and Calibration

**Figure 4:**
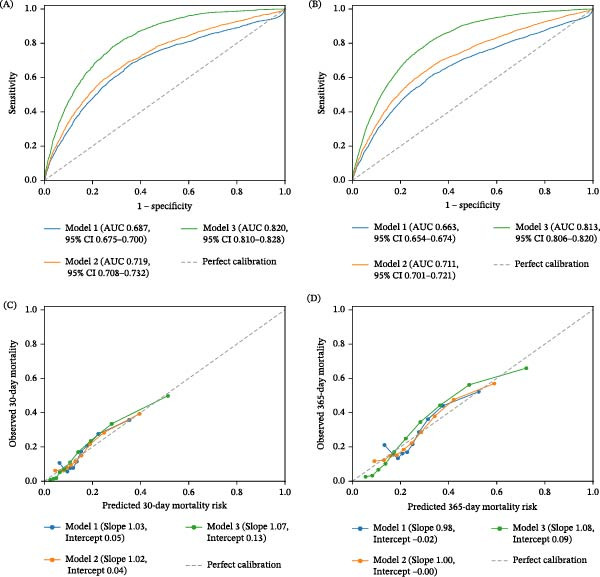
Discrimination and calibration of the Cox models for 30‐day and 365‐day all‐cause mortality. Subparts (A, B) present receiver operating characteristic curves; Subparts (C, D) present the corresponding calibration plots. Model 1 was unadjusted, Model 2 included age, sex, and race, and Model 3 used the full covariate set defined in Section 2.6.

We assessed prediction at 30 and 365 days with ROC curves and calibration plots (Figure 4). Discrimination improved with each level of adjustment. For 30‐day mortality, AUC was 0.687 (95% CI, 0.675–0.700) in Model 1, 0.719 (95% CI, 0.708–0.732) in Model 2, and 0.820 (95% CI, 0.810–0.828) in Model 3. At 365 days, AUC was 0.663 (95% CI, 0.654–0.674) in Model 1, 0.711 (95% CI, 0.701–0.721) in Model 2, and 0.813 (95% CI, 0.806–0.820) in Model 3. Calibration showed acceptable agreement between the estimated and observed risks. At 30 days, slopes were 1.03, 1.02, and 1.07, with intercepts of 0.05, 0.04, and 0.13, respectively, for Models 1–3. At 365 days, slopes were 0.98, 1.00, and 1.08, and intercepts were −0.02, 0.00, and 0.09. The curves were generally close to the ideal line. Figure S1 shows comparable findings for 90‐day and 180‐day mortality. For 90 days, AUC increased from 0.679 (95% CI, 0.668–0.690) in Model 1 to 0.719 (95% CI, 0.709–0.730) in Model 2 and 0.819 (95% CI, 0.811–0.826) in Model 3. For 180 days, AUC was 0.671 (95% CI, 0.660–0.683) in Model 1, 0.715 (95% CI, 0.706–0.727) in Model 2, and 0.817 (95% CI, 0.809–0.825) in Model 3. Model 3 therefore provided the best discrimination with acceptable calibration at every follow‐up time.

### 3.6. Subgroup Findings

**Figure 5:**
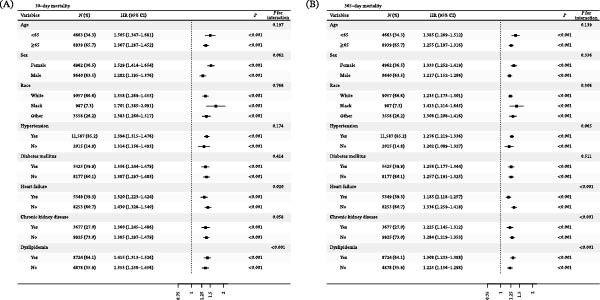
Continuous NLR and mortality within prespecified subgroups. Subparts (A, B) show the 30‐day and 365‐day endpoints, respectively. Points and horizontal bars indicate adjusted hazard ratios (HRs) and 95% confidence intervals (CIs) for log1p (NLR). Each Cox model used the Model 3 covariates except the variable defining the stratum. Interaction terms combined log1p (NLR) with the relevant subgroup indicator. The vertical dotted line denotes HR = 1.

Subgroup models evaluated the continuous log‐transformed NLR association with 30‐ and 365‐day mortality (Figure 5). Higher NLR was associated with a greater 30‐day risk in every examined stratum. Interaction was present for HF (*p* = 0.020) and dyslipidemia (*p*  < 0.001), but not for age (*p* = 0.197), sex (*p* = 0.062), race (*p* = 0.766), HTN (*p* = 0.174), DM (*p* = 0.414), or CKD (*p* = 0.058). The 365‐day association also remained significant in the subgroups. Effect modification was detected for HF, CKD, and dyslipidemia (all *p*  < 0.001). Interactions with age (*p* = 0.139), sex (*p* = 0.336), race (*p* = 0.306), HTN (*p* = 0.065), and DM (*p* = 0.511) were not significant. Thus, the direction of association was stable across major clinical strata at both follow‐up times.

### 3.7. Mediation Findings

**Figure 6:**
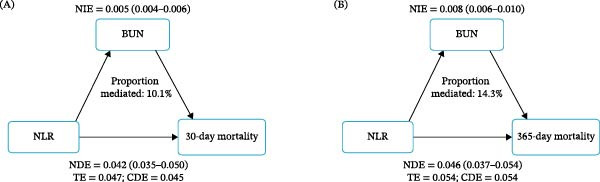
Mediation analysis of NLR, renal stress‐related biomarkers, and mortality. Subparts (A, B) correspond to the 30‐day and 365‐day endpoints, respectively. Estimates are absolute risk differences with 95% confidence intervals. CDE, controlled direct effect; NDE, natural direct effect; NIE, natural indirect effect; TE, total effect. BUN was fixed at the cohort median when estimating the CDE.

We next examined whether BUN statistically mediated the NLR‐mortality association (Figure 6). At 365 days, the natural indirect effect (NIE) was 0.008 (95% CI, 0.006–0.010), representing 14.3% of the total effect. The natural direct effect for 365‐day mortality remained 0.046 (95% CI, 0.037–0.054) after BUN was included. For 30‐day mortality, the NIE was 0.005 (95% CI, 0.004–0.006), with 10.1% being mediated. The corresponding natural direct effect for 30 day mortality was 0.042 (95% CI: 0.035–0.050). These estimates indicate partial rather than complete mediation at both time points.

### 3.8. Sensitivity Analyses

Three sets of sensitivity analyses were performed. First, an available case landmark analysis used the earliest valid NLR between 48 and 72 h after ICU admission (Table S6). NLR measured at 48–72 h remained associated with 30‐day mortality (HR, 1.159; 95% CI, 1.036–1.297; *p* = 0.010) and 365‐day mortality (HR, 1.114; 95% CI, 1.023–1.214; *p* = 0.013). Q4 at 48–72 h also carried a greater risk than Q1. In contrast, the change from 0–24 h to 48–72 h was not independently associated with either the endpoint. Second, NLR was added separately to SOFA, SAPS II, APS III, and OASIS (Table S7). AUC and C‐index improved for mortality at 30, 90, 180, and 365 days, with all delta AUC and delta C‐index *p* values  < 0.001. Third, Scr showed virtually no mediation, and eGFR showed only modest mediation (Figure S2). This contrast suggests that mediation by BUN may represent renal stress, catabolism, and neurohormonal activation rather than filtration impairment alone.

## 4. Discussion

This study showed that higher admission NLR was independently related to greater 30‐, 90‐, 180‐, and 365‐day mortality in critically ill patients with CKM Stage 4. Adjustments for demographics, acute physiology, laboratory findings, and major CKM‐related comorbidities did not materially alter the association. Its consistency in the 48–72‐h analysis, incremental value beyond ICU severity scores, and partial mediation through BUN provide complementary support. The presence of the association at four follow‐up periods indicates that the prognostic signal was not confined to early ICU mortality. Together, these results suggest that NLR captures a combination of systemic inflammation, acute physiological stress, and cardiorenal–metabolic vulnerability rather than a single isolated process.

CKM Stage 4 places established cardiovascular disease within a broader setting of kidney and metabolic dysfunction [4]. Inflammation in this setting may interact with endothelial injury, thrombotic activity, renal hypoperfusion, neurohormonal activation, and metabolic stress. Recent work in advanced CKM syndrome has also identified prognostic roles for inflammation‐ and nutrition‐related biomarkers [25]. Cardiovascular disease and CKD further share oxidative stress, vascular calcification, endothelial damage, sympathetic activation, and renin–angiotensin–aldosterone system activation [26, 27]. The NLR association should therefore not be viewed only as another generic signal of inflammation. It may instead reflect the wider cardiorenal–metabolic stress state that characterizes critical illness in CKM Stage 4. This interpretation places NLR within the integrated biology of CKM syndrome rather than within a single‐organ framework.

Both cellular components of the NLR support this interpretation. Neutrophilia can accompany innate immune activation, oxidative stress, endothelial injury, and thromboinflammation. Reactive oxygen species, proteases, and extracellular traps released by activated neutrophils may promote endothelial dysfunction, platelet activation, immunothrombosis, and vascular injury [28–31]. Stress‐related changes in neutrophil trafficking and demargination may further amplify inflammation in cardiovascular and metabolic diseases [32]. Conversely, lymphopenia may indicate neuroendocrine stress, impaired adaptive immunity, and reduced immune resilience during an acute illness [33]. A high NLR may consequently represent concurrent innate immune activation and suppression of adaptive immune capacity.

The timing of the measurement also affects the interpretation of NLR. A value obtained during the first 24 h of ICU care may reflect chronic CKM–related inflammation. It may also capture acute responses to infection, tissue injury, hypoxia, hemodynamic instability, or evolving organ dysfunction. This distinction matters because leukocyte distributions can shift rapidly during critical illness. Previous ICU studies have described NLR as a rapidly responsive marker of systemic inflammation and stress and associated it with mortality [34, 35]. In our landmark analysis, NLR measured at 48–72 h remained prognostically informative. Because this analysis was limited to patients with an available later measurement, it remains supportive rather than definitive. The latter result nevertheless reduces the concern that the primary association depended entirely on a single early measurement.

The association retained the same direction across the major clinical subgroups, although its magnitude varied in several comorbidity‐defined strata. Such variation may arise from differences in the underlying cardiorenal–metabolic context, but it does not establish causal effects within individual subgroups. Prior studies have also linked NLR to adverse outcomes in cardiovascular disease, diabetes, and CKD [36–38]. Similar prognostic or predictive associations have recently been reported in other high‐risk cardiovascular and cardiometabolic populations [39, 40]. Our results extend that evidence to a broad population with critical illness and CKM Stage 4, rather than to one narrowly defined disease category. The interactions observed in the selected comorbidity strata require confirmation and should be interpreted cautiously.

The analyses involving ICU severity scores address a separate clinical question. SOFA, SAPS II, APS III, and OASIS summarize physiological disturbance and organ dysfunction, but they do not directly measure immune‐inflammatory imbalance. Adding NLR improved the predictive performance across the mortality endpoints examined. Improvements in both AUC and C‐index support an incremental, rather than substitutive, role for NLR. This improvement does not imply that the NLR should replace an established severity score. Instead, it indicates that NLR contributes information not fully captured by conventional assessment, consistent with the expected role of an adjunctive biomarker [41, 42].

The mediation analysis offers one possible link between inflammation and renal metabolic stress. BUN accounted for part of the NLR‐mortality association at both follow‐up periods, whereas Scr showed almost no mediation and eGFR showed only a modest effect. This contrast suggests that BUN captures more than glomerular filtration alone. Its concentration is influenced by renal perfusion, neurohormonal activity, protein catabolism, volume status, corticosteroid exposure, gastrointestinal bleeding, and overall illness severity. In HF, BUN and related indices have predicted mortality and reflected hemodynamic or neurohormonal stress beyond creatinine [43–45]. The contrast between BUN and the filtration‐based markers is, therefore, clinically informative. BUN may indicate renal stress, catabolic burden, and cardiorenal instability in CKM Stage 4. However, mediation estimated from observational data cannot demonstrate a causal mechanism, so this result should be considered hypothesis‐generating [46].

From a clinical perspective, the NLR is inexpensive, rapidly available, and derived from routine blood counts. It is unlikely to be adequate as a stand‐alone predictor. Its more plausible role is to complement CKM comorbidities, renal biomarkers, and ICU severity scores. An elevated NLR may help identify patients with greater inflammatory and renal–metabolic vulnerability who merit closer monitoring and more comprehensive risk assessment. Its bedside value is likely the greatest when it is interpreted alongside comorbidities, kidney function, and the severity of acute illness.

## 5. Limitations and Future Research

Several features of the study limit its interpretation. First, its retrospective MIMIC‐IV design cannot eliminate residual confounding or information bias. Selection bias is also possible because neutrophil or lymphocyte measurements were unavailable for many otherwise eligible patients during the first 24 h. Table S8 compares included and excluded patients, but it cannot rule out nonrandom measurement. Second, CKM Stage 4 was operationalized from ICD‐9 and ICD‐10 diagnoses. This approach captures clinician‐documented disease and is common in MIMIC research, but it may miss laboratory‐defined kidney dysfunction, persistent albuminuria, adiposity patterns, or longitudinal metabolic status. Third, medication exposure could not be characterized completely. Model 3 included corticosteroids and statins, whereas limited exposure prevented the inclusion of SGLT2 inhibitors and GLP‐1 receptor agonists. Prescription records also do not fully represent prehospital treatment, adherence, timing, or indication.

Further limitations involve the measurement timing and causal interpretation. The primary exposure was based on the NLR recorded during the first 24 h of ICU care. Although the 48–72 h landmark analysis supported the main finding, it included available cases only and remains exploratory. All‐cause mortality did not distinguish cardiovascular, renal, infectious, or metabolic causes of death. The observational mediation analysis likewise cannot establish causality. Prospective multicenter studies are needed for external validation. Repeated assessment of NLR, kidney markers, inflammatory mediators, and medication exposure may refine CKM phenotyping. Such studies could determine whether their trajectories improve risk stratification in critically ill patients with CKM Stage 4.

## 6. Conclusion

In this critically ill CKM Stage 4 cohort, NLR recorded during the first 24 h of ICU care was independently related to all‐cause mortality during short‐ and long‐term follow‐up. The association was supported by subgroup and sensitivity analyses, and NLR contributed information beyond the established ICU severity scores. Partial mediation through BUN suggests a possible connection between inflammatory activity and renal metabolic stress. NLR may, therefore, be useful as an accessible adjunct, but not as a stand‐alone measure, when stratifying the risk in this population.

**Nomenclature**
AF: Atrial fibrillation
AHA: American Heart Association
ALC: Absolute lymphocyte count
AMC: Absolute monocyte count
ANC: Absolute neutrophil count
APS III: Acute Physiology Score III
AUC: Area under the curve
BMI: Body mass index
BUN: Blood urea nitrogen
CDE: Controlled direct effect
CHD: Coronary heart disease
CKD: Chronic kidney disease
CKM: Cardiovascular–kidney–metabolic
C‐index: Concordance index
CVA: Cerebrovascular disease
DBP: Diastolic blood pressure
Df: Degrees of freedom
DM: Diabetes mellitus
eGFR: Estimated glomerular filtration rate
GLP‐1 receptor agonists: Glucagon‐like peptide‐1 receptor agonists
GLU: Blood glucose
GVIF: Generalized variance inflation factor
Hb: Hemoglobin
HbA1c: Glycated hemoglobin
HF: Heart failure
HR: Hazard ratio
HTN: Hypertension
ICD: International Classification of Diseases
ICU: Intensive care unit
IHD: Ischemic heart disease
IQR: Interquartile range
MAP: Mean arterial pressure
MI: Myocardial infarction
MIMIC‐IV: Medical Information Mart for Intensive Care IV
NDE: Natural direct effect
NIE: Natural indirect effect
NLR: Neutrophil‐to‐lymphocyte ratio
OASIS: Oxford Acute Severity of Illness Score
PLT: Platelet count
PVD: Peripheral vascular disease
SBP: Systolic blood pressure
Scr: Serum creatinine
SOFA: Sequential Organ Failure Assessment
SAPS II: Simplified Acute Physiology Score II
SpO_2_: Peripheral oxygen saturation.

## Author Contributions

Longzhou Chen and Wencai Jiang conceived the study and developed its design. Yucai Tang and Hao Guo curated and collected the data. Ping Huang and Guangrong Zhang performed the statistical analyses. Longzhou Chen wrote the original draft. Xuejun Deng supervised the work and critically revised the manuscript.

## Funding

No specific funding was received for this study.

## Disclosure

All AI‐assisted wording were reviewed and revised by the authors, who take full responsibility for the final manuscript.

## Ethics Statement

The study was a secondary analysis of de‐identified MIMIC‐IV records. Institutional review boards at Beth Israel Deaconess Medical Center and the Massachusetts Institute of Technology approved the database. Additional informed consent was not required for the present analysis.

## Conflicts of Interest

The authors declare no conflicts of interest.

## Supporting Information

Additional supporting information can be found online in the Supporting Information section.

## Supporting information


**Supporting Information** The Supporting Information include eight supporting tables and two supporting figures. Table S1 describes the handling of missing data. Tables S2 and S3 compare baseline characteristics between survivors and nonsurvivors according to 90‐ and 180‐day outcomes, respectively. Table S4 presents the generalized variance inflation factors for variables included in the multivariable Cox regression Model 3. Tables S5–S7 provide additional Cox regression, sensitivity, and incremental predictive value analyses. Table S8 compares baseline characteristics between patients included in and excluded from the main NLR analysis. Figure S1 shows ROC curves and calibration plots for Cox regression models predicting 90‐ and 180‐day all‐cause mortality. Figure S2 presents sensitivity mediation analyses using serum creatinine and eGFR as alternative renal mediators.

## Data Availability

Credentialed investigators may access MIMIC‐IV through PhysioNet after completing the required training and data use agreement. Questions regarding the derived analytic cohorts may be directed to the corresponding author.
