# Supplementary material for: Prognostic Value of the Neutrophil‐to‐Lymphocyte Ratio for All‐Cause Mortality in Patients With Cardiovascular–Kidney–Metabolic Stage 4
Source: Mediators Inflamm. 2026 Jul 27;2026:9984409. doi: 10.1155/mi/9984409 (PMC13402891; doi:10.1155/mi/9984409)
Supplement: Supplementary file 1 — Supporting Information The Supporting Information include eight supporting tables and two supporting figures. Table S1 describes the handling of missing data. Tables S2 and S3 compare baseline characteristics between survivors and nonsurvivors according to 90‐ and 180‐day outcomes, respectively. Table S4 presents the generalized variance inflation factors for variables included in the multivariable Cox regression Model 3. Tables S5–S7 provide additional Cox regression, sensitivity, and incremental predictive value analyses. Table S8 compares baseline characteristics between patients included in and excluded from the main NLR analysis. Figure S1 shows ROC curves and calibration plots for Cox regression models predicting 90‐ and 180‐day all‐cause mortality. Figure S2 presents sensitivity mediation analyses using serum creatinine and eGFR as alternative renal mediators. [file MI-2026-9984409-s001.zip › Supplementary_Table_S1_Missing_data_handling.docx]

**Supplementary Table S1. Missing data handling**

| **Category** | **Variable** | **Missing, n** | **Missing, %** | **Missing-data handling** |
| --- | --- | --- | --- | --- |
| Exposure | NLR | 0 | 0.0 | Not imputed |
| Primary Cox model | Age | 0 | 0.0 | No imputation required |
| Primary Cox model | Sex | 0 | 0.0 | No imputation required |
| Primary Cox model | Race | 0 | 0.0 | No imputation required |
| Primary Cox model | Heart rate | 13 | 0.1 | Random forest-based multiple imputation using Python scikit-learn |
| Primary Cox model | Systolic blood pressure | 13 | 0.1 | Random forest-based multiple imputation using Python scikit-learn |
| Primary Cox model | Diastolic blood pressure | 13 | 0.1 | Random forest-based multiple imputation using Python scikit-learn |
| Primary Cox model | White blood cell count | 0 | 0.0 | Random forest-based multiple imputation using Python scikit-learn |
| Primary Cox model | Hemoglobin | 3 | 0.0 | Random forest-based multiple imputation using Python scikit-learn |
| Primary Cox model | Platelet count | 2 | 0.0 | Random forest-based multiple imputation using Python scikit-learn |
| Primary Cox model | Blood urea nitrogen | 8 | 0.1 | Random forest-based multiple imputation using Python scikit-learn |
| Primary Cox model | Serum creatinine | 5 | 0.0 | Random forest-based multiple imputation using Python scikit-learn |
| Primary Cox model | Blood glucose | 130 | 1.0 | Random forest-based multiple imputation using Python scikit-learn |
| Primary Cox model | Sodium | 12 | 0.1 | Random forest-based multiple imputation using Python scikit-learn |
| Primary Cox model | Potassium | 28 | 0.2 | Random forest-based multiple imputation using Python scikit-learn |
| Primary Cox model | Hypertension | 0 | 0.0 | No imputation required |
| Primary Cox model | Diabetes mellitus | 0 | 0.0 | No imputation required |
| Primary Cox model | Chronic kidney disease | 0 | 0.0 | No imputation required |
| Primary Cox model | Atrial fibrillation | 0 | 0.0 | No imputation required |
| Primary Cox model | Heart failure | 0 | 0.0 | No imputation required |
| Primary Cox model | Myocardial infarction | 0 | 0.0 | No imputation required |
| Primary Cox model | Ischemic heart disease | 0 | 0.0 | No imputation required |
| Primary Cox model | Cerebrovascular disease | 0 | 0.0 | No imputation required |
| Primary Cox model | Peripheral vascular disease | 0 | 0.0 | No imputation required |
| Sensitivity analyses | SOFA | 0 | 0.0 | No imputation required |
| Sensitivity analyses | SAPS II | 0 | 0.0 | No imputation required |
| Sensitivity analyses | APS III | 0 | 0.0 | No imputation required |
| Sensitivity analyses | OASIS | 0 | 0.0 | No imputation required |
| Sensitivity analyses | Mechanical ventilation | 0 | 0.0 | No imputation required |
| Sensitivity analyses | Renal replacement therapy | 0 | 0.0 | No imputation required |
| Sensitivity analyses | Vasopressor use | 0 | 0.0 | No imputation required |
| Primary multivariable model | Corticosteroids | 0 | 0.0 | No imputation required; included in Model 3 |
| Primary multivariable model | Statins | 0 | 0.0 | No imputation required; included in Model 3 |
| Sparse medication variables | SGLT2 inhibitors | 0 | 0.0 | Descriptive only; not imputed |
| Sparse medication variables | GLP-1 receptor agonists | 0 | 0.0 | Descriptive only; not imputed |
| Excluded candidate variables | Height | 4123 | 30.3 | Excluded from multivariable analyses; not imputed |
| Excluded candidate variables | BMI | 4135 | 30.4 | Excluded from multivariable analyses; not imputed |
| Excluded candidate variables | Triglycerides | 12245 | 90.0 | Excluded from multivariable analyses; not imputed |
| Excluded candidate variables | HbA1c | 12250 | 90.1 | Excluded from multivariable analyses; not imputed |
| Excluded candidate variables | Albumin | 9168 | 67.4 | Excluded from multivariable analyses; not imputed |
| Excluded candidate variables | Lactate | 3705 | 27.2 | Excluded from multivariable analyses; not imputed |
| Excluded candidate variables | C-reactive protein | 13030 | 95.8 | Excluded from multivariable analyses; not imputed |

For covariates with less than 20% missingness, random forest-based multiple imputation was performed in Python using the scikit-learn package. Five imputed datasets were generated using different random seeds, and regression estimates were pooled according to Rubin’s rules. The exposure variable, survival time, and mortality outcomes were not imputed.
