# Supplementary material for: Prognostic Value of the Neutrophil‐to‐Lymphocyte Ratio for All‐Cause Mortality in Patients With Cardiovascular–Kidney–Metabolic Stage 4
Source: Mediators Inflamm. 2026 Jul 27;2026:9984409. doi: 10.1155/mi/9984409 (PMC13402891; doi:10.1155/mi/9984409)
Supplement: Supplementary file 1 — Supporting Information The Supporting Information include eight supporting tables and two supporting figures. Table S1 describes the handling of missing data. Tables S2 and S3 compare baseline characteristics between survivors and nonsurvivors according to 90‐ and 180‐day outcomes, respectively. Table S4 presents the generalized variance inflation factors for variables included in the multivariable Cox regression Model 3. Tables S5–S7 provide additional Cox regression, sensitivity, and incremental predictive value analyses. Table S8 compares baseline characteristics between patients included in and excluded from the main NLR analysis. Figure S1 shows ROC curves and calibration plots for Cox regression models predicting 90‐ and 180‐day all‐cause mortality. Figure S2 presents sensitivity mediation analyses using serum creatinine and eGFR as alternative renal mediators. [file MI-2026-9984409-s001.zip › Supplementary_Table_S3_180_day_survival_status.docx]

**Supplementary Table S3. Comparisons between survivors and non-survivors based on 180-day outcomes**

| **Characteristics** | **Overall (n = 13,602)** | **Survivors (n = 10,511)** | **Non-survivors (n = 3,091)** | **P value** |
| --- | --- | --- | --- | --- |
| Age (years) | 70.00 (61.00 - 78.00) | 68.00 (60.00 - 77.00) | 75.00 (66.00 - 83.00) | <0.001 |
| Sex (%) |  |  |  | <0.001 |
| Female | 4,962 (36.48%) | 3,597 (34.22%) | 1,365 (44.16%) |  |
| Male | 8,640 (63.52%) | 6,914 (65.78%) | 1,726 (55.84%) |  |
| Race (%) |  |  |  | <0.001 |
| White | 9,057 (66.59%) | 7,083 (67.39%) | 1,974 (63.86%) |  |
| Black | 987 (7.26%) | 733 (6.97%) | 254 (8.22%) |  |
| Other | 3,558 (26.16%) | 2,695 (25.64%) | 863 (27.92%) |  |
| NLR | 6.63 (3.95 - 12.03) | 5.94 (3.72 - 10.02) | 10.84 (5.76 - 20.73) | <0.001 |
| NLR quartile (%) |  |  |  | <0.001 |
| Q1 | 3,401 (25.00%) | 2,925 (27.83%) | 476 (15.40%) |  |
| Q2 | 3,400 (25.00%) | 2,968 (28.24%) | 432 (13.98%) |  |
| Q3 | 3,400 (25.00%) | 2,613 (24.86%) | 787 (25.46%) |  |
| Q4 | 3,401 (25.00%) | 2,005 (19.08%) | 1,396 (45.16%) |  |
| HR (beats/min) | 82.00 (74.00 - 95.00) | 80.00 (74.00 - 91.00) | 90.00 (76.00 - 106.00) | <0.001 |
| SBP (mmHg) | 117.00 (104.00 - 134.00) | 117.00 (104.00 - 133.00) | 119.00 (102.00 - 137.00) | 0.327 |
| DBP (mmHg) | 63.00 (54.00 - 74.00) | 62.00 (54.00 - 73.00) | 65.00 (53.00 - 78.00) | <0.001 |
| RR (breaths/min) | 18.00 (15.00 - 22.00) | 17.00 (15.00 - 20.00) | 20.00 (17.00 - 25.00) | <0.001 |
| SpO2 (%) | 99.00 (96.00 - 100.00) | 99.00 (96.00 - 100.00) | 97.00 (94.00 - 100.00) | <0.001 |
| ANC (K/uL) | 9.27 (6.35 - 13.04) | 9.05 (6.34 - 12.44) | 10.10 (6.37 - 15.41) | <0.001 |
| ALC (K/uL) | 1.32 (0.80 - 2.01) | 1.46 (0.93 - 2.15) | 0.89 (0.52 - 1.42) | <0.001 |
| WBC (K/uL) | 11.70 (8.40 - 15.90) | 11.50 (8.40 - 15.40) | 12.30 (8.30 - 18.10) | <0.001 |
| Hb (g/dL) | 10.10 (8.60 - 11.60) | 10.00 (8.60 - 11.60) | 10.10 (8.60 - 11.80) | 0.101 |
| PLT (K/uL) | 165.00 (122.00 - 227.00) | 162.00 (122.00 - 217.00) | 185.00 (121.00 - 262.00) | <0.001 |
| BUN (mg/dL) | 20.00 (14.00 - 32.00) | 18.00 (14.00 - 27.00) | 31.00 (20.00 - 51.00) | <0.001 |
| Scr (mg/dL) | 1.00 (0.80 - 1.50) | 1.00 (0.80 - 1.30) | 1.40 (0.90 - 2.30) | <0.001 |
| GLU (mg/dL) | 126.00 (107.00 - 158.00) | 124.00 (106.00 - 152.00) | 138.00 (109.00 - 188.00) | <0.001 |
| Na (mmol/L) | 138.00 (136.00 - 141.00) | 138.00 (136.00 - 141.00) | 138.00 (135.00 - 142.00) | 0.535 |
| K (mmol/L) | 4.30 (3.90 - 4.70) | 4.30 (3.90 - 4.60) | 4.30 (3.80 - 4.80) | 0.173 |
| SOFA | 5.00 (3.00 - 7.00) | 5.00 (3.00 - 7.00) | 6.00 (4.00 - 10.00) | <0.001 |
| SAPS II | 38.00 (31.00 - 47.00) | 36.00 (29.00 - 43.00) | 48.00 (39.00 - 59.00) | <0.001 |
| APS III | 41.00 (31.00 - 57.00) | 37.00 (29.00 - 50.00) | 57.00 (44.00 - 76.00) | <0.001 |
| OASIS | 32.00 (27.00 - 38.00) | 31.00 (26.00 - 37.00) | 37.00 (31.00 - 44.00) | <0.001 |
| HTN (%) | 11,587 (85.19%) | 8,999 (85.62%) | 2,588 (83.73%) | 0.01 |
| DM (%) | 5,425 (39.88%) | 4,172 (39.69%) | 1,253 (40.54%) | 0.411 |
| CKD (%) | 3,677 (27.03%) | 2,532 (24.09%) | 1,145 (37.04%) | <0.001 |
| Obesity (%) | 2,935 (21.58%) | 2,247 (21.38%) | 688 (22.26%) | 0.307 |
| Dyslipidemia (%) | 8,724 (64.14%) | 7,151 (68.03%) | 1,573 (50.89%) | <0.001 |
| AF (%) | 6,414 (47.15%) | 4,726 (44.96%) | 1,688 (54.61%) | <0.001 |
| HF (%) | 5,349 (39.33%) | 3,775 (35.91%) | 1,574 (50.92%) | <0.001 |
| MI (%) | 3,587 (26.37%) | 2,770 (26.35%) | 817 (26.43%) | 0.949 |
| IHD (%) | 8,784 (64.58%) | 7,110 (67.64%) | 1,674 (54.16%) | <0.001 |
| CVD (%) | 2,324 (17.09%) | 1,590 (15.13%) | 734 (23.75%) | <0.001 |
| PVD (%) | 2,320 (17.06%) | 1,728 (16.44%) | 592 (19.15%) | <0.001 |
| Mechanical ventilation (%) | 8,938 (65.71%) | 7,093 (67.48%) | 1,845 (59.69%) | <0.001 |
| RRT (%) | 726 (5.34%) | 354 (3.37%) | 372 (12.03%) | <0.001 |
| Vasopressor use (%) | 6,930 (50.95%) | 5,386 (51.24%) | 1,544 (49.95%) | 0.215 |
| Corticosteroid use (%) | 2,747 (20.20%) | 1,638 (15.58%) | 1,109 (35.88%) | <0.001 |
| Statin use (%) | 9,518 (69.98%) | 7,987 (75.99%) | 1,531 (49.53%) | <0.001 |

## NLR, neutrophil-to-lymphocyte ratio; HR, heart rate; SBP, systolic blood pressure; DBP, diastolic blood pressure; RR, respiratory rate; SpO₂, peripheral oxygen saturation; ANC, absolute neutrophil count; ALC, absolute lymphocyte count; WBC, white blood cell count; Hb, hemoglobin; PLT, platelet count; BUN, blood urea nitrogen; Scr, serum creatinine; GLU, blood glucose; Na, serum sodium; K, serum potassium; SOFA, Sequential Organ Failure Assessment; SAPS II, Simplified Acute Physiology Score II; APS III, Acute Physiology Score III; OASIS, Oxford Acute Severity of Illness Score; HTN, hypertension; DM, diabetes mellitus; CKD, chronic kidney disease; AF, atrial fibrillation; HF, heart failure; MI, myocardial infarction; IHD, ischemic heart disease; CVD, cerebrovascular disease; PVD, peripheral vascular disease; RRT, renal replacement therapy.
