# Supplementary material for: Prognostic Value of the Neutrophil‐to‐Lymphocyte Ratio for All‐Cause Mortality in Patients With Cardiovascular–Kidney–Metabolic Stage 4
Source: Mediators Inflamm. 2026 Jul 27;2026:9984409. doi: 10.1155/mi/9984409 (PMC13402891; doi:10.1155/mi/9984409)
Supplement: Supplementary file 1 — Supporting Information The Supporting Information include eight supporting tables and two supporting figures. Table S1 describes the handling of missing data. Tables S2 and S3 compare baseline characteristics between survivors and nonsurvivors according to 90‐ and 180‐day outcomes, respectively. Table S4 presents the generalized variance inflation factors for variables included in the multivariable Cox regression Model 3. Tables S5–S7 provide additional Cox regression, sensitivity, and incremental predictive value analyses. Table S8 compares baseline characteristics between patients included in and excluded from the main NLR analysis. Figure S1 shows ROC curves and calibration plots for Cox regression models predicting 90‐ and 180‐day all‐cause mortality. Figure S2 presents sensitivity mediation analyses using serum creatinine and eGFR as alternative renal mediators. [file MI-2026-9984409-s001.zip › Supplementary_Table_S4_GVIF_Model3.docx]

**Supplementary Table S4. Generalized variance inflation factors (GVIFs) for variables included in the multivariable Cox regression Model 3**

| Variable | Df | GVIF | GVIF^(1/(2*Df)) |
| --- | --- | --- | --- |
| NLR | 1 | 1.1583 | 1.0763 |
| Age | 1 | 1.2991 | 1.1398 |
| Sex | 1 | 1.1574 | 1.0758 |
| Race | 2 | 1.1075 | 1.0259 |
| HR | 1 | 1.2102 | 1.1001 |
| SBP | 1 | 1.6293 | 1.2764 |
| DBP | 1 | 1.788 | 1.3372 |
| WBC | 1 | 1.0443 | 1.0219 |
| HGB | 1 | 1.2147 | 1.1021 |
| PLT | 1 | 1.1267 | 1.0615 |
| BUN | 1 | 2.2246 | 1.4915 |
| Scr | 1 | 2.1644 | 1.4712 |
| Blood glucose | 1 | 1.1497 | 1.0722 |
| Sodium | 1 | 1.0905 | 1.0443 |
| Potassium | 1 | 1.1611 | 1.0775 |
| HTN | 1 | 1.0353 | 1.0175 |
| DM | 1 | 1.1769 | 1.0848 |
| CKD | 1 | 1.4292 | 1.1955 |
| Obesity | 1 | 1.0614 | 1.0302 |
| Dyslipidemia | 1 | 1.1971 | 1.0941 |
| AF | 1 | 1.1432 | 1.0692 |
| HF | 1 | 1.1634 | 1.0786 |
| MI | 1 | 1.2995 | 1.14 |
| IHD | 1 | 1.6015 | 1.2655 |
| CVA | 1 | 1.0875 | 1.0429 |
| PVD | 1 | 1.0237 | 1.0118 |
| Corticosteroids | 1 | 1.1012 | 1.0494 |
| Statins | 1 | 1.4025 | 1.1843 |

GVIF, generalized variance inflation factor; Df, degrees of freedom; HR, heart rate; SBP, systolic blood pressure; DBP, diastolic blood pressure; HGB, hemoglobin; PLT, platelet count; Body Mass Index; WBC, white blood cell count; Scr, serum creatinine; BUN, blood urea nitrogen; PVD, peripheral vascular disease; AF, atrial fibrillation; DM, diabetes mellitus; HTN, hypertension; CVA, cerebrovascular accident; CKD, chronic kidney disease; HF, heart failure; MI, myocardial infarction; IHD, ischemic heart disease; NLR, neutrophil-to-lymphocyte ratio.
