# Supplementary material for: Prognostic Value of the Neutrophil‐to‐Lymphocyte Ratio for All‐Cause Mortality in Patients With Cardiovascular–Kidney–Metabolic Stage 4
Source: Mediators Inflamm. 2026 Jul 27;2026:9984409. doi: 10.1155/mi/9984409 (PMC13402891; doi:10.1155/mi/9984409)
Supplement: Supplementary file 1 — Supporting Information The Supporting Information include eight supporting tables and two supporting figures. Table S1 describes the handling of missing data. Tables S2 and S3 compare baseline characteristics between survivors and nonsurvivors according to 90‐ and 180‐day outcomes, respectively. Table S4 presents the generalized variance inflation factors for variables included in the multivariable Cox regression Model 3. Tables S5–S7 provide additional Cox regression, sensitivity, and incremental predictive value analyses. Table S8 compares baseline characteristics between patients included in and excluded from the main NLR analysis. Figure S1 shows ROC curves and calibration plots for Cox regression models predicting 90‐ and 180‐day all‐cause mortality. Figure S2 presents sensitivity mediation analyses using serum creatinine and eGFR as alternative renal mediators. [file MI-2026-9984409-s001.zip › Supplementary_Table_S5_Cox_models_90_day_and_180_day.docx]

**Supplementary Table S5. Cox proportional hazards models for 90-day and 180-day all-cause mortality according to NLR quartiles**

| Events (%) | | Model 1 |  | Model 2 |  | Model 3 |  |
| --- | --- | --- | --- | --- | --- | --- | --- |
|  |  | HR (95% CI) | P value | HR (95% CI) | P value | HR (95% CI) | P value |
| 90-day mortality |  |  |  |  |  |  |  |
| Q1(n=3,401) | 394 (11.6) | 1 |  | 1 |  | 1 |  |
| Q2(n=3,400) | 356 (10.5) | 0.899 (0.779-1.037) | 0.145 | 0.919 (0.797-1.061) | 0.249 | 0.917 (0.793-1.059) | 0.238 |
| Q3(n=3,400) | 689 (20.3) | 1.845 (1.630-2.088) | <0.001 | 1.787 (1.579-2.023) | <0.001 | 1.417 (1.248-1.610) | <0.001 |
| Q4(n=3,401) | 1,262 (37.1) | 3.777 (3.374-4.229) | <0.001 | 3.458 (3.086-3.874) | <0.001 | 1.969 (1.745-2.223) | <0.001 |
| P for trend |  |  | <0.001 |  | <0.001 |  | <0.001 |
| 180-day mortality |  |  |  |  |  |  |  |
| Q1(n=3,401) | 476 (14.0) | 1 |  | 1 |  | 1 |  |
| Q2(n=3,400) | 432 (12.7) | 0.902 (0.792-1.027) | 0.12 | 0.925 (0.812-1.053) | 0.238 | 0.914 (0.802-1.043) | 0.181 |
| Q3(n=3,400) | 787 (23.1) | 1.757 (1.568-1.969) | <0.001 | 1.702 (1.519-1.907) | <0.001 | 1.339 (1.191-1.505) | <0.001 |
| Q4(n=3,401) | 1,396 (41.0) | 3.545 (3.195-3.933) | <0.001 | 3.244 (2.922-3.602) | <0.001 | 1.841 (1.647-2.058) | <0.001 |
| P for trend |  |  | <0.001 |  | <0.001 |  | <0.001 |

Model 1 included NLR; Model 2 was additionally adjusted for age, sex, and race; Model 3 was further adjusted for heart rate, systolic blood pressure, diastolic blood pressure, white blood cell count, hemoglobin, platelet count, blood urea nitrogen, serum creatinine, blood glucose, sodium, potassium, hypertension, diabetes mellitus, chronic kidney disease, obesity, dyslipidemia, atrial fibrillation, heart failure, myocardial infarction, ischemic heart disease, cerebrovascular disease, peripheral vascular disease, corticosteroid use, and statin use.
