# Supplementary material for: Prognostic Value of the Neutrophil‐to‐Lymphocyte Ratio for All‐Cause Mortality in Patients With Cardiovascular–Kidney–Metabolic Stage 4
Source: Mediators Inflamm. 2026 Jul 27;2026:9984409. doi: 10.1155/mi/9984409 (PMC13402891; doi:10.1155/mi/9984409)
Supplement: Supplementary file 1 — Supporting Information The Supporting Information include eight supporting tables and two supporting figures. Table S1 describes the handling of missing data. Tables S2 and S3 compare baseline characteristics between survivors and nonsurvivors according to 90‐ and 180‐day outcomes, respectively. Table S4 presents the generalized variance inflation factors for variables included in the multivariable Cox regression Model 3. Tables S5–S7 provide additional Cox regression, sensitivity, and incremental predictive value analyses. Table S8 compares baseline characteristics between patients included in and excluded from the main NLR analysis. Figure S1 shows ROC curves and calibration plots for Cox regression models predicting 90‐ and 180‐day all‐cause mortality. Figure S2 presents sensitivity mediation analyses using serum creatinine and eGFR as alternative renal mediators. [file MI-2026-9984409-s001.zip › Supplementary_Table_S6_48_72_hour_and_dynamic_NLR_sensitivity.docx]

**Supplementary Table S6. Sensitivity analyses using 48-72-hour NLR and dynamic changes in NLR for 30-day and 365-day all-cause mortality**

| Outcome | Analysis | Exposure | N | Events | HR (95% CI) | P value | HR | Lower95CI | Upper95CI | P |
| --- | --- | --- | --- | --- | --- | --- | --- | --- | --- | --- |
| 30-day mortality | 48-72h NLR, continuous | ln(NLR48+1), per 1-unit increase | 1228 | 323 | 1.159 (1.036-1.297) | 0.01 | 1.159 | 1.036 | 1.297 | 0.010 |
| 30-day mortality | 48-72h NLR quartiles | Q2 vs Q1 | 1228 | 323 | 0.822 (0.582-1.161) | 0.266 | 0.822 | 0.582 | 1.161 | 0.266 |
| 30-day mortality | 48-72h NLR quartiles | Q3 vs Q1 | 1228 | 323 | 1.089 (0.789-1.502) | 0.606 | 1.089 | 0.789 | 1.502 | 0.606 |
| 30-day mortality | 48-72h NLR quartiles | Q4 vs Q1 | 1228 | 323 | 1.416 (1.043-1.924) | 0.026 | 1.416 | 1.043 | 1.924 | 0.026 |
| 30-day mortality | Dynamic NLR, continuous | Delta NLR, per 1-SD increase | 1228 | 323 | 0.951 (0.860-1.052) | 0.327 | 0.951 | 0.860 | 1.052 | 0.327 |
| 30-day mortality | Dynamic NLR increase | Increase vs no increase | 1228 | 323 | 0.888 (0.710-1.111) | 0.298 | 0.888 | 0.710 | 1.111 | 0.298 |
| 30-day mortality | P for trend across 48-72h NLR quartiles | Quartile ordinal trend | 1228 | 323 | 1.176 (1.047-1.321) | 0.006 | 1.176 | 1.047 | 1.321 | 0.006 |
| 30-day mortality | 48-72h NLR quartile events | Q1 | 307 | 72 |  |  |  |  |  |  |
| 30-day mortality | 48-72h NLR quartile events | Q2 | 307 | 56 |  |  |  |  |  |  |
| 30-day mortality | 48-72h NLR quartile events | Q3 | 307 | 85 |  |  |  |  |  |  |
| 30-day mortality | 48-72h NLR quartile events | Q4 | 307 | 110 |  |  |  |  |  |  |
| 365-day mortality | 48-72h NLR, continuous | ln(NLR48+1), per 1-unit increase | 1228 | 561 | 1.114 (1.023-1.214) | 0.013 | 1.114 | 1.023 | 1.214 | 0.013 |
| 365-day mortality | 48-72h NLR quartiles | Q2 vs Q1 | 1228 | 561 | 1.047 (0.814-1.346) | 0.722 | 1.047 | 0.814 | 1.346 | 0.722 |
| 365-day mortality | 48-72h NLR quartiles | Q3 vs Q1 | 1228 | 561 | 1.166 (0.909-1.494) | 0.226 | 1.166 | 0.909 | 1.494 | 0.226 |
| 365-day mortality | 48-72h NLR quartiles | Q4 vs Q1 | 1228 | 561 | 1.317 (1.035-1.675) | 0.025 | 1.317 | 1.035 | 1.675 | 0.025 |
| 365-day mortality | Dynamic NLR, continuous | Delta NLR, per 1-SD increase | 1228 | 561 | 1.032 (0.952-1.120) | 0.441 | 1.032 | 0.952 | 1.120 | 0.441 |
| 365-day mortality | Dynamic NLR increase | Increase vs no increase | 1228 | 561 | 1.080 (0.912-1.280) | 0.371 | 1.080 | 0.912 | 1.280 | 0.371 |
| 365-day mortality | P for trend across 48-72h NLR quartiles | Quartile ordinal trend | 1228 | 561 | 1.115 (1.021-1.217) | 0.016 | 1.115 | 1.021 | 1.217 | 0.016 |
| 365-day mortality | 48-72h NLR quartile events | Q1 | 307 | 130 |  |  |  |  |  |  |
| 365-day mortality | 48-72h NLR quartile events | Q2 | 307 | 123 |  |  |  |  |  |  |
| 365-day mortality | 48-72h NLR quartile events | Q3 | 307 | 145 |  |  |  |  |  |  |
| 365-day mortality | 48-72h NLR quartile events | Q4 | 307 | 163 |  |  |  |  |  |  |

NLR48 was defined as the first valid neutrophil-to-lymphocyte ratio measured between 48 and 72 hours after ICU admission. Dynamic NLR was defined as the difference between NLR48 and the first available NLR within 0–24 hours after ICU admission. Cox proportional hazards models were fitted using a landmark design, with follow-up starting from the time of NLR48 measurement. Models were adjusted for the covariates included in Model 3. Continuous NLR48 was analyzed as ln(NLR48 + 1), and dynamic NLR was analyzed per 1-SD increase. HR, hazard ratio; CI, confidence interval; NLR, neutrophil-to-lymphocyte ratio.
