# Supplementary material for: Prognostic Value of the Neutrophil‐to‐Lymphocyte Ratio for All‐Cause Mortality in Patients With Cardiovascular–Kidney–Metabolic Stage 4
Source: Mediators Inflamm. 2026 Jul 27;2026:9984409. doi: 10.1155/mi/9984409 (PMC13402891; doi:10.1155/mi/9984409)
Supplement: Supplementary file 1 — Supporting Information The Supporting Information include eight supporting tables and two supporting figures. Table S1 describes the handling of missing data. Tables S2 and S3 compare baseline characteristics between survivors and nonsurvivors according to 90‐ and 180‐day outcomes, respectively. Table S4 presents the generalized variance inflation factors for variables included in the multivariable Cox regression Model 3. Tables S5–S7 provide additional Cox regression, sensitivity, and incremental predictive value analyses. Table S8 compares baseline characteristics between patients included in and excluded from the main NLR analysis. Figure S1 shows ROC curves and calibration plots for Cox regression models predicting 90‐ and 180‐day all‐cause mortality. Figure S2 presents sensitivity mediation analyses using serum creatinine and eGFR as alternative renal mediators. [file MI-2026-9984409-s001.zip › Supplementary_Table_S7_Incremental_predictive_value.docx]

**Supplementary Table S7. Incremental predictive value of NLR beyond established ICU severity scores for all-cause mortality**

| **Outcome** | **Severity score** | **Score alone AUC**  **(95% CI)** | **Score + NLR AUC**  **(95% CI)** | **Delta AUC**  **(95% CI)** | **P for Delta AUC** | **Score alone C-index**  **(95% CI)** | **Score + NLR C-index**  **(95% CI)** | **Delta C-index**  **(95% CI)** | **P for Delta C-index** | **N** | **Events** |
| --- | --- | --- | --- | --- | --- | --- | --- | --- | --- | --- | --- |
| 30-day mortality | SOFA | 0.676 (0.662-0.691) | 0.741 (0.729-0.754) | 0.065 (0.055-0.074) | <0.001 | 0.669 (0.656-0.681) | 0.727 (0.716-0.737) | 0.058 (0.049-0.067) | <0.001 | 13602 | 2063 |
| 30-day mortality | SAPS II | 0.771 (0.761-0.782) | 0.797 (0.788-0.807) | 0.026 (0.020-0.032) | <0.001 | 0.757 (0.747-0.768) | 0.780 (0.770-0.789) | 0.023 (0.017-0.028) | <0.001 | 13602 | 2063 |
| 30-day mortality | APS III | 0.786 (0.775-0.796) | 0.803 (0.793-0.812) | 0.017 (0.011-0.022) | <0.001 | 0.770 (0.760-0.780) | 0.785 (0.776-0.794) | 0.014 (0.009-0.020) | <0.001 | 13602 | 2063 |
| 30-day mortality | OASIS | 0.728 (0.716-0.741) | 0.766 (0.754-0.778) | 0.037 (0.030-0.045) | <0.001 | 0.715 (0.705-0.727) | 0.748 (0.738-0.759) | 0.033 (0.026-0.040) | <0.001 | 13602 | 2063 |
| 90-day mortality | SOFA | 0.649 (0.636-0.661) | 0.718 (0.707-0.729) | 0.069 (0.060-0.079) | <0.001 | 0.643 (0.631-0.654) | 0.704 (0.693-0.714) | 0.061 (0.053-0.069) | <0.001 | 13602 | 2701 |
| 90-day mortality | SAPS II | 0.756 (0.746-0.767) | 0.780 (0.771-0.790) | 0.024 (0.019-0.030) | <0.001 | 0.740 (0.730-0.748) | 0.761 (0.751-0.769) | 0.021 (0.016-0.027) | <0.001 | 13602 | 2701 |
| 90-day mortality | APS III | 0.771 (0.761-0.780) | 0.785 (0.776-0.794) | 0.014 (0.009-0.020) | <0.001 | 0.753 (0.744-0.761) | 0.765 (0.756-0.773) | 0.012 (0.008-0.017) | <0.001 | 13602 | 2701 |
| 90-day mortality | OASIS | 0.702 (0.690-0.713) | 0.742 (0.731-0.752) | 0.040 (0.033-0.047) | <0.001 | 0.690 (0.681-0.699) | 0.724 (0.715-0.734) | 0.034 (0.028-0.040) | <0.001 | 13602 | 2701 |
| 180-day mortality | SOFA | 0.636 (0.624-0.647) | 0.706 (0.695-0.716) | 0.070 (0.062-0.080) | <0.001 | 0.631 (0.621-0.641) | 0.692 (0.682-0.702) | 0.061 (0.052-0.069) | <0.001 | 13602 | 3091 |
| 180-day mortality | SAPS II | 0.748 (0.739-0.758) | 0.771 (0.761-0.781) | 0.023 (0.018-0.028) | <0.001 | 0.731 (0.722-0.739) | 0.751 (0.742-0.758) | 0.020 (0.015-0.025) | <0.001 | 13602 | 3091 |
| 180-day mortality | APS III | 0.761 (0.752-0.770) | 0.774 (0.766-0.783) | 0.013 (0.008-0.018) | <0.001 | 0.743 (0.734-0.751) | 0.754 (0.746-0.762) | 0.011 (0.007-0.016) | <0.001 | 13602 | 3091 |
| 180-day mortality | OASIS | 0.693 (0.682-0.704) | 0.732 (0.721-0.742) | 0.039 (0.033-0.046) | <0.001 | 0.680 (0.671-0.690) | 0.714 (0.706-0.723) | 0.034 (0.028-0.039) | <0.001 | 13602 | 3091 |
| 365-day mortality | SOFA | 0.615 (0.605-0.626) | 0.689 (0.679-0.699) | 0.073 (0.064-0.083) | <0.001 | 0.614 (0.604-0.623) | 0.676 (0.667-0.684) | 0.062 (0.055-0.069) | <0.001 | 13602 | 3620 |
| 365-day mortality | SAPS II | 0.731 (0.721-0.741) | 0.754 (0.745-0.763) | 0.023 (0.018-0.028) | <0.001 | 0.715 (0.707-0.723) | 0.734 (0.726-0.742) | 0.020 (0.015-0.024) | <0.001 | 13602 | 3620 |
| 365-day mortality | APS III | 0.750 (0.740-0.759) | 0.761 (0.753-0.770) | 0.011 (0.006-0.017) | <0.001 | 0.731 (0.723-0.738) | 0.741 (0.733-0.748) | 0.010 (0.005-0.014) | <0.001 | 13602 | 3620 |
| 365-day mortality | OASIS | 0.673 (0.664-0.683) | 0.714 (0.705-0.725) | 0.041 (0.035-0.048) | <0.001 | 0.663 (0.655-0.672) | 0.698 (0.690-0.706) | 0.035 (0.029-0.040) | <0.001 | 13602 | 3620 |

AUC and C-index were calculated to evaluate the incremental prognostic value of NLR when added to established ICU severity score models, including SOFA, SAPS II, APS III, and OASIS. Outcomes included 30-day, 90-day, 180-day, and 365-day all-cause mortality. Delta AUC and delta C-index represent the differences between the severity score alone model and the corresponding severity score plus NLR model. P values for delta AUC and delta C-index were estimated using paired bootstrap comparisons. NLR, neutrophil-to-lymphocyte ratio; AUC, area under the receiver operating characteristic curve; C-index, concordance index; SOFA, Sequential Organ Failure Assessment; SAPS II, Simplified Acute Physiology Score II; APS III, Acute Physiology Score III; OASIS, Oxford Acute Severity of Illness Score; CI, confidence interval.
